# Cardioprotective Melatonin: Translating from Proof-of-Concept Studies to Therapeutic Use

**DOI:** 10.3390/ijms20184342

**Published:** 2019-09-05

**Authors:** Ovidiu Constantin Baltatu, Sergio Senar, Luciana Aparecida Campos, José Cipolla-Neto

**Affiliations:** 1 Center of Innovation, Technology and Education (CITE), School of Health Sciences at Anhembi Morumbi University, Laureate International Universities, Sao Jose dos Campos 12247-016, Brazil; ocbaltatu@gmail.com (O.C.B) (L.A.C.); 2 DrTarget, 28806 Madrid, Spain; 3 Department of Physiology and Biophysics, Institute of Biomedical Sciences, University of São Paulo, São Paulo 05508-900, Brazil

**Keywords:** melatonin, cardioprotection, cardiovascular system, drugs, machine learning, in silico

## Abstract

In this review we summarized the actual clinical data for a cardioprotective therapeutic role of melatonin, listed melatonin and its agonists in different stages of development, and evaluated the melatonin cardiovascular target tractability and prediction using machine learning on ChEMBL. To date, most clinical trials investigating a cardioprotective therapeutic role of melatonin are in phase 2a. Selective melatonin receptor agonists Tasimelteon, Ramelteon, and combined melatonergic-serotonin Agomelatine, and other agonists with registered structures in CHEMBL were not yet investigated as cardioprotective or cardiovascular drugs. As drug-able for these therapeutic targets, melatonin receptor agonists have the benefit over melatonin of well-characterized pharmacologic profiles and extensive safety data. Recent reports of the X-ray crystal structures of MT1 and MT2 receptors shall lead to the development of highly selective melatonin receptor agonists. Predictive models using machine learning could help to identify cardiovascular targets for melatonin. Selecting ChEMBL scores > 4.5 in cardiovascular assays, and melatonin scores > 4, we obtained 284 records from 162 cardiovascular assays carried out with 80 molecules with predicted or measured melatonin activity. Melatonin activities (agonistic or antagonistic) found in these experimental cardiovascular assays and models include arrhythmias, coronary and large vessel contractility, and hypertension. Preclinical proof-of-concept and early clinical studies (phase 2a) suggest a cardioprotective benefit from melatonin in various heart diseases. However, larger phase 3 randomized interventional studies are necessary to establish melatonin and its agonists’ actions as cardioprotective therapeutic agents.

## 1. Introduction

**Figure 1:**
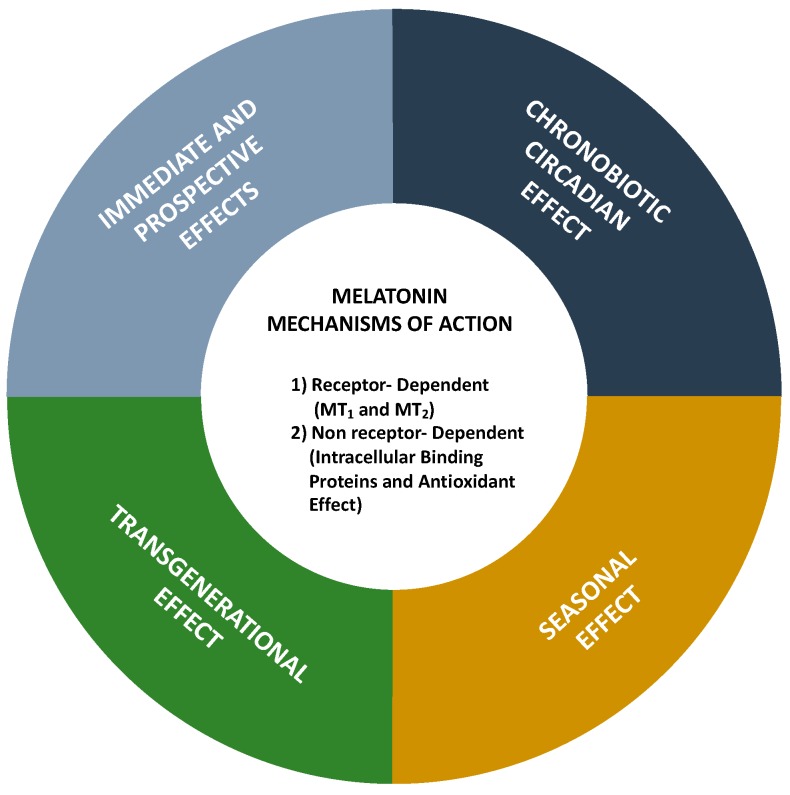
Melatonin mechanisms of action.

Melatonin (*N*-acetyl-5-methoxytryptamine) is an indolamine with a molecular mass of 232.283 g/mol that presents two basic properties: (1) It is amphiphilic (partition coefficient of 1.2), being almost equally diffusible in aqueous and in lipid medium so that it can be found in all compartments of the organism, and (2) it is a potent antioxidant molecule, efficiently scavenging almost any oxygen and nitrogen reactive species. Its origin, and supposedly its primary function, seems to be linked to this antioxidant potency that is well preserved throughout the phylogenetic tree. The other melatonin cellular and systemic functions and also different ways of action were acquired during evolution, according to the complexity of the organism and the site of melatonin production [1,2]. Synthesized from the amino acid tryptophan as the primary substrate, in complex organisms, in mammals, melatonin can be produced by several peripheral tissues for local use (autocrine/paracrine action), besides being produced by the pineal gland and released in the circulation, acting as a hormone. Melatonin’s mechanisms of actions are mediated through receptor-dependent and non-receptor dependent (intracellular binding proteins and antioxidant effects) pathways (Figure 1). Melatonin acts as a biological time–domain molecule acting on the circadian, seasonal, and transgenerational timescales (Figure 1).

There are two specific melatonin membrane receptors, nominated MT1 (or MTNR1A) and MT2 (MTRN1B). They are protein-coupled receptors linked to Gi/Go or Gq/G11, and signaling, therefore, happens either through the inhibition of cAMP or cGMP synthesis or activation of phospholipase C, depending on the central or peripheral localization of the target. These melatonin receptors are ubiquitously present in peripheral and central organs, including the cardiovascular system. MTNR1B mutations are associated with increased blood sugar levels and increased risk for type 2 diabetes, while variants in MTNR1A and B have been linked to autism spectrum disorders [3,4]. Due to its phylogenetic origin and its amphiphilicity, melatonin can also act in a nonreceptor-mediated way, regulating directly some intracellular biochemical processes. In this case, in addition to direct scavenging free radicals and free radical products, melatonin can interact with molecular effectors, such as the enzyme calcium/calmodulin-dependent kinase II. It should be pointed out that the well-known antioxidant effect of melatonin is, in part, dependent on its direct interaction with reactive species of oxygen and nitrogen, besides increasing receptor-mediated antioxidant expression.

Melatonin controls cardiovascular function (blood pressure and heart rate) acting either peripherally (heart and blood vessels) or centrally (hypothalamus, area postrema, caudal ventrolateral medulla, and/or the rostral ventrolateral medulla), in addition to regulating the renin-angiotensin system [5] and mitochondrial function [6].

A large preclinical portfolio suggests a cardioprotective role and other cardiovascular benefits of melatonin. Evidence for cardiovascular benefits of dietary melatonin has been recently reviewed [7]. Due to its pleiotropic actions on the cardiovascular system, melatonin has gained therapeutic interest for cardiac and cardiovascular patients [8].

In this review, we summarized the actual clinical data for a cardioprotective therapeutic role of melatonin, listed melatonin and its agonists in different stages of development, and evaluated the melatonin cardiovascular target tractability and prediction using machine learning on ChEMBL.

## 2. Clinical Trials with Melatonin in Heart Diseases

### 2.1. Acute Coronary Syndrome

Acute coronary syndrome (ACS) contains three types of coronary artery disease: ST-segment elevation myocardial infarction (STEMI), non–ST-segment elevation myocardial infarction (NSTEMI), and unstable angina. Reasons for the use of melatonin in acute coronary syndrome have been reviewed by Dominguez-Rodriguez et al. [9,10]. Stipulated mechanisms for benefic effects melatonin in this pathology include vasodilatory effects of vascular melatoninergic receptors, antioxidative and anti-inflammatory actions, and influence on circadian cardiovascular rhythms [9,10]. Through these mechanisms, several preclinical studies have suggested melatonin as effective in reducing ischemia-reperfusion injury, including injury in the heart (reviewed by Reiter and Tan, [11]).

Although intravenous and intracoronary melatonin during primary percutaneous coronary intervention (PPCI) in patients with STEMI was not associated with a reduction in infarct size (Clinical trials: NCT00640094) [12], a significant effect appeared in a post hoc analysis for patients who presented early after symptom onset [13] (Table 1).

Results are awaited from the following completed investigational randomized trials:

Completed trial on prophylactic melatonin treatment for preventive effect on depression, depressive and anxiety symptoms, sleep, and circadian disturbances following acute coronary syndrome (Clinical trials: NCT02451293) [20].

Completed trial on intracoronary and systemic melatonin effects on the Myocardial Salvage Index, high-sensitivity troponin, creatine kinase myocardial band, and clinical events have been investigated in patients with acute myocardial infarction (Clinical trials: NCT01172171) [21].

An ongoing study aims at researching the cardioprotective effects of intravenous melatonin administered prior to reperfusion and continued after restoration of coronary blood flow in patients with ST-segment elevation myocardial infarction undergoing primary percutaneous coronary intervention (Clinical trials: NCT03303378).

### 2.2. Coronary Artery Disease

Melatonin 5 mg in nondippers with coronary artery disease decreased nocturnal blood pressure but also caused a daytime increase [22]. Stipulated mechanisms for the beneficial effects of melatonin in this pathology include an influence on circadian cardiovascular rhythms of blood pressure. Effects of melatonin on progression of coronary artery calcification are being investigated in an ongoing randomized trial (Clinical trials: NCT03966235). The investigators of this study reason that several preclinical studies have shown that melatonin protects against inflammation and apoptosis in vascular calcification (Clinical trials: NCT03966235). However, we were unable to find any published report on melatonin and vascular or coronary artery calcification.

### 2.3. Cardiac Arrhythmias

A clinical intervention trial is underway to evaluate whether melatonin decreases the occurrence of atrial fibrillation after cardiac surgery (Clinical trials: NCT02099331), based on its reported putative antiarrhythmic effects preclinical experimental models [23,24,25]. A putative antiarrhythmic effect of melatonin has been implicated to occur in preclinical studies by reducing sympathetic tone [26] and its antioxidant activity [27]. In the meantime, a clinical case report suggests that melatonin can potentially induce ventricular arrhythmias [28]. Therefore, the ultimate proarrhythmic vs. antiarrhythmic effect of melatonin awaits to be further clarified in translational studies [28].

### 2.4. Heart Failure

Preclinical studies have documented the beneficial effects of melatonin in reducing myocardial tissue injury in models of experimental ischemia-reperfusion [29,30]. Furthermore, the beneficial effects of melatonin in heart failure might be through its effects on cardiovascular health, blood pressure, and endothelial function.

An ongoing trial studies the effect of melatonin 10 mg on cardiovascular and muscle mass and function in patients with heart failure (Clinical trials: NCT03894683).

Melatonin treatment in the perioperative period of major surgery (50 mg melatonin infusion over a 2 h period intraoperatively, and 10 mg melatonin orally the first three nights after surgery) decreased clinical cardiac morbidity and the occurrence of myocardial ischemia after abdominal aortic aneurism repair (Clinical trials: NCT00315926) [16].

### 2.5. Hypertension

The first indications for melatonin involvement in blood pressure regulations date back in early 70 s when pinealectomy-induced hypertension that could be prevented with melatonin was described in rats [31,32]. Mechanisms involved could include all presented earlier in this review, but most of the clinical studies are counting on the melatonin’s influence on circadian cardiovascular rhythms of blood pressure. Hypertension with circadian alterations of blood pressure, a common cause of cardiac complications, has been investigated as a therapeutic target for melatonin. Randomized controlled trials with hypertensive patients reported effects on the following outcome measures:

Decrease in systolic blood pressure throughout the 24 h period by melatonin (5 mg oral, 4 weeks) [33].

Increase in 24-h mean blood pressure by melatonin (5 mg oral, 4 weeks treatment) in hypertensive patients treated by nifedipine [34].

Decrease in nocturnal blood pressure in patients with untreated essential hypertension by repeated (3 weeks) but not single-dose melatonin (2.5 mg) [35].

Decrease in nocturnal blood pressure in normotensive and treated hypertensive women by melatonin sustained-release (1 mg rapidly and 2 mg slowly) [36].

Decrease in nocturnal systolic blood pressure in patients with nocturnal hypertension by melatonin sustained-release (2 mg for 4 weeks) [37]

Improvement in sleep quality in hypertensive patients treated with beta-blockers by melatonin (2.5 mg for 3 weeks) (ClinicalTrials: NCT00238108) [38].

No significant effect on nocturnal blood pressure in African Americans with essential hypertension by melatonin 24 mg sustained-release was found (Clinical trials: NCT01114373) [39].

A meta-analysis of melatonin effects on nocturnal blood pressure concluded that controlled-release melatonin is effective and safe in ameliorating nocturnal hypertension [40]. Though, the summed up number of patients from these trials was low (149 subjects for fast-release melatonin, 72 subjects for controlled-release melatonin). Also, the dosage was not taken into account.

The conclusions from these trials are awaiting to be reinforced with further phase 2b studies to confirm the clinical efficacy of melatonin and determine the therapeutic dose range. Different study populations and time-dependent covariates like sleep apnea should be considered. Further phase 3 randomized controlled trials are necessary to confirm and expand on safety and effectiveness the results from phase 2 trials.

### 2.6. Melatonin Receptor Agonists 

Pharmacokinetic parameters of melatonin in humans have been reviewed by Harpsøe NG et al. [41]. No link between specific melatonin plasma concentration levels and actual clinical effects (or adverse effects) has been established yet. Doses of 0.3 to 100 mg of melatonin are typically administered orally, sublingually, or intravenously. Pharmacokinetic parameters, indications, dosing, and common adverse events of commercially available melatonin receptor agonists were summarized by Williams WP 3rd et al. [42]. Common adverse events include nausea, dizziness, and somnolence.

Twelve melatonin receptor small molecules agonists were identified in the ChEMBL bioactivity database, which has > 2 million compound records created by mining and extracting data from medicinal chemistry literature [43] (Table 2).

**Tasimelteon**, a selective dual MT1 and MT2 melatonin receptor agonist, was not investigated in heart or cardiovascular disorders. Tasimelteon is indicated for the treatment of non-24-h sleep-wake disorder (N24HSWD) [44,45], and it is also effective for transient insomnia after sleep-time shift ClinicalTrials: NCT00490945 and NCT00291187) [46]. Tasimelteon was investigated for the treatment of depressive disorder (Clinical trials: NCT01428661), Smith-Magenis syndrome (Clinical trials: NCT02231008), liver diseases (Clinical trials: NCT01271387), without conclusive (published) results.

**Ramelteon**, a selective dual MT1 and MT2 melatonin receptor agonist, was cardioprotective through postconditioning in a preclinical heart ischemia-reperfusion injury model proof-of-concept study [47]. The cardioprotection induced by Ramelteon is melatonin receptor-dependent and operates through activation of mKCa and mKATP channels [48]. To date, there are no clinical reports of Ramelteon’s efficacy on cardiac or cardiovascular diseases. Ramelteon is indicated for the treatment of insomnia [49]. Ramelteon was ineffective for the treatment of bipolar disorder (Clinical trials: NCT01467713) [50].

**Agomelatine**, (CHEMBL10878) is a melatonergic agonist (at both MT1 and MT2 receptors) and serotonin 2C (5-HT2C) receptor antagonist indicated for the treatment of major depression [51].

Melatonin analogs CHEMBL3230568, CHEMBL3230569, CHEMBL34730, CHEMBL33700 [52], melatonergic ligands CHEMBL498494, CHEMBL498493, CHEMBL525374 [53], selective melatonin MT1 and MT2 agonists CHEMBL15060, CHEMBL34730, CHEMBL33415 [54,55,56,57,58] were reviewed by Zlotos et al. [59].

Two recent papers elucidated the crystal structures and characterized the structural basis of ligand recognition for the human MT1 [60] and MT2 [61] melatonin receptors. MT1 was characterized in complex with four agonists: Ramelteon, two melatonin analogs (CHEMBL15060—2-phenylmelatonin and CHEMBL289233—2-iodomelatonin), and agomelatine (mixed melatonin-serotonin agonist). The MT2 combined with ramelteon and CHEMBL15060 (melatonin analog—2-phenylmelatonin). The X-ray crystal structures of MT1 and MT2 receptors represent a milestone in melatonin research that will facilitate the design of highly selective melatonin lead compounds and therapeutic agents.

## 3. Evaluation of Melatonin Cardiovascular Target Tractability and Prediction Using Machine Learning on ChEMBL

**Figure 2:**
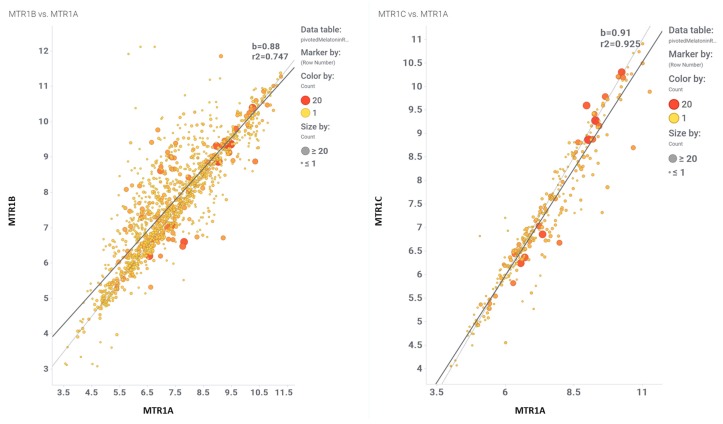
Comparison of the melatonin potency score for MTR1B vs. MTR1A, and MTR1 vs. MTR1A.

The fundamental step in drug discovery is the generation of a working hypothesis to justify a new lead compound for a therapeutic target. Machine learning models as virtual assays are widely used for compound target prediction and quantitative structure-activity relationship predictions [62]. The ChEMBL database is the largest primary open data source of manually extracted and curated structure-activity relationship data [63,64]. The ChEMBL database contains 5 k records of functional and binding melatonin experiments belonging to 356 different assays carried out in MTR1A, MTR1B, and MTR1C target proteins using 1617 different molecules (Supplement file S1, ‘melatoninChemblAssaysUsed’ Excel sheet) in 110 citations (Supplement file S2, ‘CHEMBL25-chembl_document-aA2WyI’ Excel sheet). A melatonin potency score was calculated for them all. Melatonin potency score is a transformation of the ChEMBL values of activity because they are recorded with different dimensions into a value similar to −log(potency) in molar scale, which is required to make potency comparisons consistent. Given the high correlation between the three receptor MTR1A, MTR1B, and MTR1C subtypes, an average melatonin potency score was used for machine learning purposes (Figure 2, and Supplement file S1, ‘activitiesRecordedInChemblByMTR’ Excel sheet). For these 1617 compounds, there are 17 k records from > 5 k experimental protocols carried out in a plethora of target-based and phenotypic assays. These records were used as the signature to build our validation set. A final step was the addition of melatonin negative bioactivity data because prediction of activity requires the existence of a population of non-active records [65]. As among the 1617 melatonin related molecules the population of non-active records is almost missing, this was created by adding a 60 k members artificial population from molecules being inactive in at least 10 GPCR assays. The final recorded interactions of our validation set, actives plus autogenerated inactives reached 1.5 M data. Variables included in the validation set are related to chemical properties, assay properties, target properties, taxonomy, cell lines, tissues or organs, and some enumerated variables to allow development of random forest algorithms. The validation set incorporated 60 variables describing each experimental fact besides a categorical classification of melatonin activity and a melatonin activity score.

Evaluation of predictive models.

**Figure 3:**
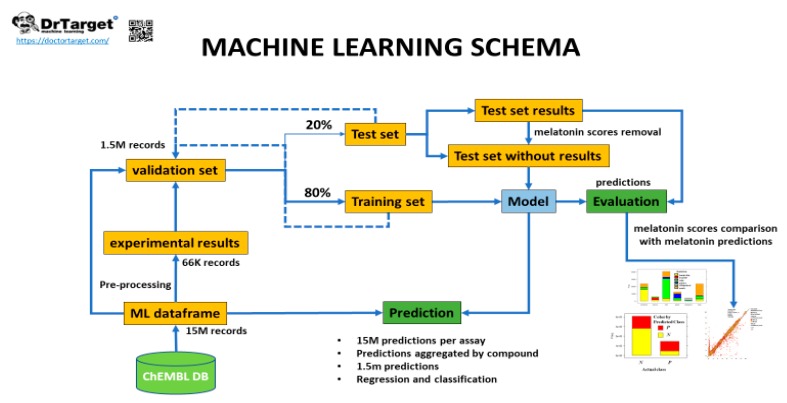
Machine learning schema for melatonin activity.

**Figure 4:**
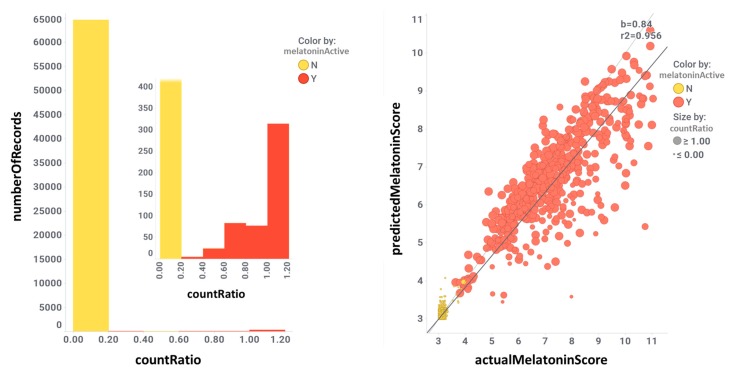
Correlation for regression between the actual and predicted melatonin activity scores (**right**) and the count ratio. Color is by actual classification label (red = active, yellow = inactive). CountRatio bar chart inset is the same chart zooming in the active zone.

**Figure 5:**
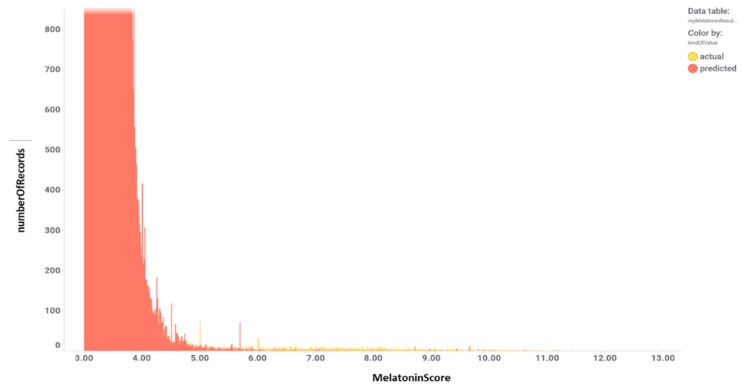
Activity distribution histogram for the 1.5 M compounds.

**Figure 6:**
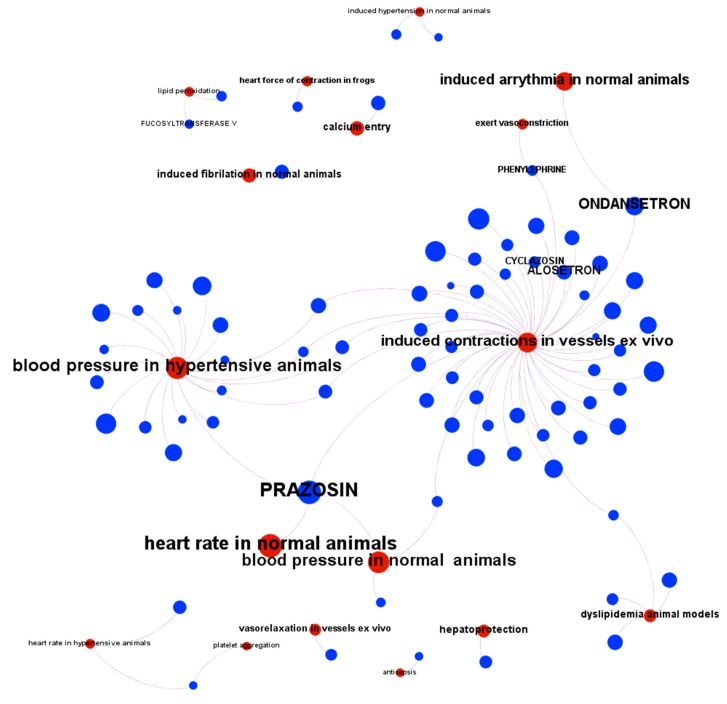
Diagram of the interaction of predicted melatonin active molecules on different models of cardiovascular assays. In red: CV assay model, blue: molecule. When the molecule has a drug name, this name is displayed. When the molecule has a simple registry number it is hidden to allow better visualization.

The validation set was split in an 80/20 ratio, i.e., training and test sets, respectively (Figure 3). The training set was used to generate random forest classification and regression models [66]. The test set was then passed through the model once the actual melatonin score and labels were removed. This generated a prediction of melatonin prediction upon the 20% of the validation set records that could be compared to the actual values previously removed, thus allowing prediction quality evaluation. Predictions were then aggregated by compound so that an average activity score by compound was calculated. For classification model, a parameter called countRatio was calculated: basically, the ratio between the occasions a compound is classified active vs. total occasions it appears in the validation set. Figure 4 shows the correlation for regression between the actual and predicted melatonin activity scores (right) and the count ratio. Now, the model can be applied to the whole database records (15 M) and aggregated for the 1.5 M compounds. This is technically a virtual screening campaign. In this step, the calculated melatonin score is a combination of countRatio and melatonin prediction score so that this combination is applied only when no actual score exists. Figure 5 shows the activity distribution histogram for the 1.5 M compounds. If we consider active those with a score > 4 we have got 9 k hits. Two hundred fifty-four (254) compounds that have a drug name were identified as melatonin active (Supplement file S1, ‘drugsIdentifiedAsMelatActive’ Excel sheet). In order to see what cardiovascular assays are associated, we used the three main parameters: (1) The melatonin score already defined based on the actual or predicted potency score measured or calculated for melatonin activity; (2) the ChEMBL score, the measured potency score of a molecule in a particular ChEMBL assay; (3) the number of molecules that each particular assay has seen. Selecting ChEMBL scores > 4.5 in cardiovascular assays, and melatonin scores > 4, we obtained 284 records from 162 cardiovascular assays carried out with 80 molecules with predicted or measured melatonin activity (Supplement file S1, 284 records are divided in two Excel sheets: 162 records for in vivo assays ‘inVivoCVassaysWithMelatoninComp’ and 122 records for in vitro assays ‘inVitroCVassaysWithMelatoninCom’). In Figure 6 the range of the experimental models used in these assays is visualized. Melatonin activities (agonistic or antagonistic) found in these experimental cardiovascular assays and models include arrhythmias, coronary and large vessel contractility, hypertension, and interactions with prazosin and ondansetron.

## 4. Conclusions and Further Directions

Melatonin, a multifunctional indoleamine, has attributed cardioprotective role through several mechanisms, and apparently has a safe pharmacological profile. Despite the vast preclinical evidence for a cardioprotective and cardiovascular protective melatonin, a better understanding of the pharmacokinetics and pharmacodynamics of melatonin and its associated pharmacophores shall help to translate the basic research findings into a clinical setting.

Melatonin is marketed as a dietary food supplement and commonly prescribed for insomnia [67]. It has not been investigated yet in large-scale, long-term phase 2b and 3 clinical trials. A role of melatonin and its agonists’ actions as cardioprotective therapeutic agents shall be established in larger phase 3 randomized interventional studies.

Melatonin receptor agonists have the benefit over melatonin of well-characterized pharmacologic profiles and selective action [42]. Unraveling the X-ray crystal structures of MT1 and MT2 receptors shall lead to the development of highly selective melatonin receptor agonists. Predictive models using machine learning could help in identifying cardiovascular targets for melatonin.

## Figures and Tables

**Table 1:** Clinical trials with melatonin in heart diseases.

Target Disease, Reference	Objective	Study Phase	Study Design	Melatonin dosing	Outcomes
Myocardial Infarction [12]	Melatonin Adjunct in the Acute myocaRdial Infarction Treated With Angioplasty (MARIA)/NCT00640094	2	randomized, double-blind, placebo controlled trial	Intravenous and intracoronary melatonin during primary percutaneous coronary intervention (PPCI).	Negative: melatonin in patients with STEMI was not associated with a reduction in infarct size and has an unfavourable effect on the ventricular volumes and LVEF evolution
ST-Elevation Myocardial Infarction (STEMI) [13]	Post hoc of MARIA study/NCT00640094	2	randomized, double-blind, placebo controlled trial		Positive: melatonin in patients with STEMI who presented early after symptom onset was associated with a significant reduction in the infarct size after pPCI
Coronary artery bypass grafting [14]	Efficacy of melatonin in reducing early reperfusion injury and acute oxidative stress in patients undergoing coronary artery bypass grafting (CABG).	2	randomized, open-label, placebo-controlled trial		Positive: melatonin significantly reduced CABG related cardiac injury and oxidative stress.
ST-Elevation Myocardial Infarction (STEMI) [15]	To study whether the administration of melatonin during acute myocardial reperfusion improves myocardial salvage assessed by cardiac magnetic resonance imaging (CMR) in patients with STEMI	2	randomized, double-blinded, placebo controlled trial	intracoronary or intravenous melatonin (total 50 mg)	Negative: melatonin did not improve the myocardial salvage index.
Elective abdominal aortic aneurism repair [16]	To study the effect of perioperative melatonin treatment on clinical cardiac morbidity and markers of myocardial ischemia in patients undergoing elective surgery for abdominal aortic aneurism		randomized, placebo-controlled, clinical trial	infusion over a 2-hr period either, 50 mg melatonin or placebo intra-operatively, and 10 mg melatonin or placebo orally, the first three nights after surgery.	Positive: melatonin decreased clinical cardiac morbidity and the occurrence of myocardial ischemia after abdominal aortic aneurism repair.
Postural tachycardia syndrome (POTS) [17]	Tested the hypothesis that melatonin will attenuate the tachycardia and improve symptom burden in patients with POTS. NCT00262470	2	randomized, single-blinded, crossover trial	melatonin 3 mg orally and placebo, on separate mornings, in a randomized crossover design	Negative: There was no significant difference in the reduction of systolic blood pressure between melatonin and placebo, either with standing or while seated. The symptom burden was not improved with melatonin compared with placebo.
Coronary artery bypass grafting surgery (CABG) [18]	To investigate the effects of Melatoninon nuclear erythroid 2-related factor 2(Nrf2) activity in patients undergoing CABG surgery	2	randomized triple-blind placebo-controlled trial	10 mg oral melatonin (Melatonin group, *n* = 15) or placebo (placebo group, *n* = 15) before sleeping for 1 month before surgery	Positive: Increase by melatonin of nuclear erythroid 2-related factor 2(Nrf2) activity in patients undergoing CABG surgery.
Blood coagulation activity [19]	To investigate if oral administration of melatonin is associated with decreased plasma levels of procoagulant hemostatic measures	2	randomized, placebo-controlled, single-blinded trial	3 mg of oral melatonin or placebo, and one hour thereafter, levels of melatonin, fibrinogen, and D-dimer as well as activities of coagulation factor VII (FVII:C) and VIII (FVIII:C) were measured in plasma	Positive: lower levels of the coagulation measures FVIII:C and fibrinogen one hour after oral intake of a single dose of 3 mg of melatonin compared to placebo medication. Suggested potential implications for the use of melatonin as a theapeutic agent in patients at-risk of atherothrombotic events such as patients with CAD or systemic hypertension

**Table 2:** ChEMBL Melatonin Agonists. ‘ChEMBL ID’ = The externally viewed identification for each compound, ‘Max Phase’ = phase of clinical development, ‘QED Weighted’ = quantitative estimate of drug-likeness, ‘mesh heading’ = Medical subject heading (MeSH) assigned to the citation by National Library of Medicine (NLM) indexing, ‘melatoninScore’ = melatonin potency score is a transformation of the ChEMBL values of activity because they are recorded with different dimensions into a value similar to −log(potency) in molar scale, which is required to make potency comparisons consistent.

ChEMBL ID	Name	Synonyms	Max Phase	Molecular Weight	QED Weighted	Mesh_Heading
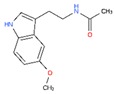 CHEMBL45	MELATONIN	Circadin, General Nutrit, Health Aid, Heidadouppi, Icenia, Life Ext, Melapure, Melatonin, *N*-(2-(5-Methoxy-1H-Indol-3-Yl)Ethyl)Acetamide, *N*-Acetyl-5-Methoxytryptamine, Natrol, Nature’s Blend, Natures Bounty, Quality Health, S.Gard, Travelag, Vespro, Vytalonin	4	232.28	0.84	Anxiety, Aortic Aneurysm, Atrial Fibrillation, Attention Deficit Disorder with Hyperactivity, Autistic Disorder, Barrett Esophagus, Brain Ischemia, Breast Neoplasms, Carcinoma, Non-Small-Cell Lung, Child Development Disorders, Pervasive, Cognitive Dysfunction, Colitis, Ulcerative, Dementia, Depressive Disorder, Dermatitis, Atopic, Diabetes Mellitus, Epilepsy, Fatigue, Fibromyalgia, Gastroesophageal Reflux, Head and Neck Neoplasms, Hemorrhage, Hypertension, Melanoma, Metabolic Diseases, Metabolic Syndrome, Migraine Disorders, Multiple Sclerosis, Relapsing-Remitting, Myocardial Infarction, Neonatal Sepsis, Neoplasms, Nocturnal Enuresis, Pain, Premature Birth, Reperfusion Injury, Schizophrenia, Sepsis, Skin Diseases, Sleep Initiation and Maintenance Disorders, Stomatitis, Substance Withdrawal Syndrome, Sunburn
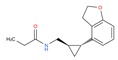 CHEMBL2103822	TASIMELTEON	BMS-214778, Hetlioz, Tasimelteon, VEC-162	4	245.32	0.88	Depressive Disorder, Liver Diseases, Sleep Initiation and Maintenance Disorders, Smith-Magenis Syndrome
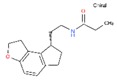 CHEMBL1218	RAMELTEON	Ramelteon, Rozerem, TAK-375	4	259.35	0.9	Bipolar Disorder, Depressive Disorder, Marijuana Abuse, Migraine with Aura, Migraine without Aura, Pulmonary Disease, Chronic Obstructive, Sleep Apnea, Obstructive, Sleep Initiation and Maintenance Disorders, Substance-Related Disorders, Tobacco Use Disorder
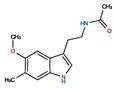 CHEMBL3230568	No Data		0	246.31	0.87	
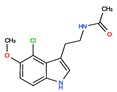 CHEMBL3230569	No Data		0	266.73	0.89	
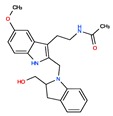 CHEMBL498494	No Data		0	393.49	0.58	
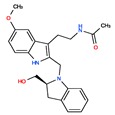 CHEMBL498493	No Data		0	393.49	0.58	
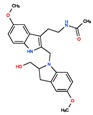 CHEMBL525374	No Data		0	423.51	0.52	
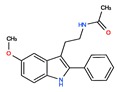 CHEMBL15060	No Data		0	308.38	0.76	
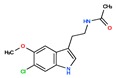 CHEMBL34730	No Data	6-Chloromelatonin	0	266.73	0.89	
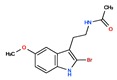 CHEMBL33415	No Data		0	311.18	0.91	
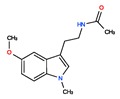 CHEMBL33700	No Data		0	246.31	0.89	

## Supplementary Materials

Supplementary materials can be found at https://www.mdpi.com/1422-0067/20/18/4342/s1.
